# Supplementary figures and images for: Sex differences in saliva-based DNA methylation changes and environmental stressor in young African American adults
Source: PLoS One. 2022 Sep 6;17(9):e0273717. doi: 10.1371/journal.pone.0273717 (PMC9447871; doi:10.1371/journal.pone.0273717)

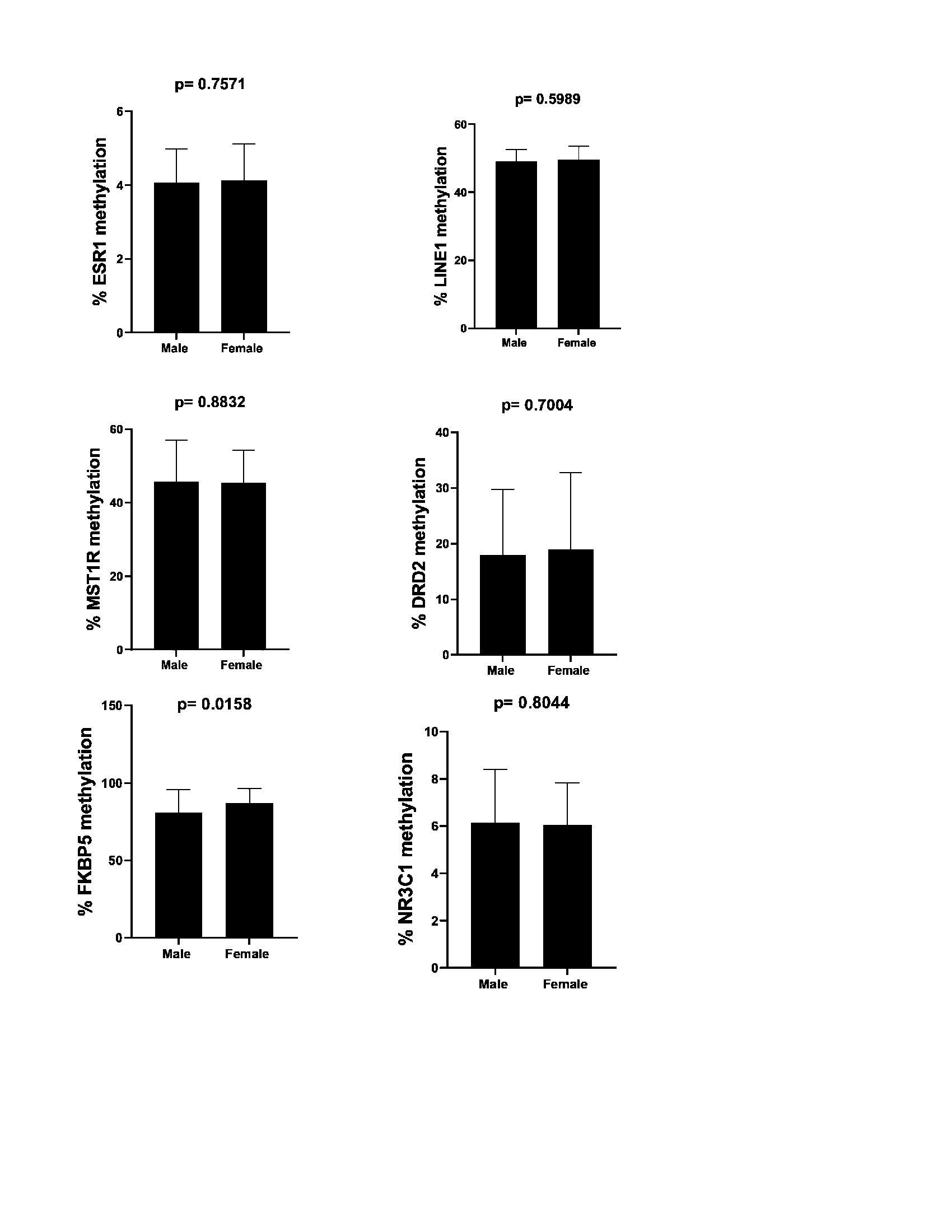

Supplement: S1 Fig — Methylation levels stratified by sex shows significant difference between males and females for FKBP5 (p = 0.015; Fisher T-test). (JPG) [file pone.0273717.s001.jpg]
