# Supplementary material for: Sex differences in saliva-based DNA methylation changes and environmental stressor in young African American adults
Source: PLoS One. 2022 Sep 6;17(9):e0273717. doi: 10.1371/journal.pone.0273717 (PMC9447871; doi:10.1371/journal.pone.0273717)
Supplement: S1 Table — (DOCX) [file pone.0273717.s002.docx]

| **Gene** | **Pyrosequencing primers and sequence to analyze** | **CpG sites** | **PCR conditions** | **Amplicon Size (bp)** | **Nucleotide position from TSS (bp)** |
| --- | --- | --- | --- | --- | --- |
| LINE1 | F (5’-3’): TTTTGAGTTAGGTGTGGGATATA  R(5’-3’): Biotin-AAAATCAAAAAATTCCCTTTC  Sequencing primer: AGTTAGGTGTGGGATATAGT  Sequence to analyze (anti-sense strand): TT**Y**GTGGTG**Y**GGT**Y**GTTTTTTAAGT**Y**GGTTTGAAAAG | 4 | -95^o^C for 5 minutes  50 cycles of  -95^o^C for 30 seconds  - 56^o^C for 30 seconds  -72^o^C for 30 seconds  Final 72^o^c for 7 minutes | 146 bp | -347 |
| ESR1 | F (5’-3’): TGTGTTTTTTTTTTAGGTGG  R (5’-3’): Biotin-AACCATCCCAAATACTTTAATA  Sequencing primer: GGATACGGTTTGTATTTTG  Sequence to analyze (sense strand):  TT**Y**G**Y**GGTTA**Y**GGATTATGATTATGATTTTTTATATTAAAGTATTTGGGATGGTT | 3 | -95^o^C for 5 minutes  50 cycles of  -95^o^C for 30 seconds  - 56^o^C for 30 seconds  -72^o^C for 45 seconds  Final 72^o^c for 7 minutes | 125 bp | +226 |
| MST1R | F (5’-3’): TTTTAGTTTTTTAAATTGTTGGGATTATAG  R (5’-3’): Biotin-GGGACACCGCTGATCGTTTACCTTCCTCTAACACCATCTTAT  Sequencing primer: AAATTGTTGGGATTATAGG  Sequence to analyze (sense strand):  **Y**GTAAGTTAT**Y**G**Y**GTT**Y**GGT**Y**GGGATTATTTTTTTATGTGTTTTTTT | 5 | -95^o^C for 5 minutes  50 cycles of  -95^o^C for 30 seconds  - 56^o^C for 30 seconds  -72^o^C for 45 seconds  Final 72^o^C for 7 minutes | 169 bp | +155 |
| NR3C1 | F (5’-3’): Biotin- 5’AATTTTTTAGGAAAAAGGGTGG  R (5’-3’): AACCCCTTTCCAAATAACACACTTC  Sequencing primer: AACTCCCCAATAAATCTAAAAC  Sequence to analyze (anti-sense strand):  C**R**C**R**AAACTAAAC**R**AAAAC**R**AAAAAAAAATAAC | 4 | -95^o^C for 5 minutes  50 cycles of  -95^o^C for 30 seconds  - 60^o^C for 30 seconds  -72^o^C for 30 seconds  Final 72^o^C for 7 minutes | 32 bp | -500 |
| FKBP5  (Intron 7) | F (5’-3’): GGATTTGTAGTTGGGATAATAATTTGG  R (5’-3’): Biotin-TCTTACCTCCAACACTACTACTAAAA  Sequencing primer: GGAGTTATAGTGTAGGTTT  Sequence to analyze (sense strand): TTT**Y**GTGATTTTTGTGAAGGGTATA ATT**Y**GTTTAGTTTTGAAAAG | 2 | -95^o^C for 5 minutes  50 cycles of  -95^o^C for 30 seconds  - 60^o^C for 30 seconds  -72^o^C for 30 seconds  Final 72^o^C for 7 minutes | 81 bp | +15099 |
| DRD2 | F (5’-3’): TTAGGTAAGGAGAGGAGGTGTT  R (5’-3’): Biotin- ACTAAAATCCAAAACCTTAAATACATCA  Sequencing primer: AGGAGGTGTTGGAAG  Sequence to analyze: AGGTT**Y**GTATT**Y**GTTTTTTGATTTTAGGTT TTGGAATT | 2 | -95^o^C for 5 minutes  50 cycles of  -95^o^C for 30 seconds  - 54^o^C for 30 seconds  -72^o^C for 30 seconds  Final 72^o^C for 7 minutes | 91 | 1500 |

Supplementary Table 1S.

Forward (F) and Reverse (R) and sequencing primers are shown for each gene promoter assay. Pyrosequencing information of sequence for analysis is shown including CpG (C/T) polymorphic sites; shown in bold red as Y or R for sense strand or antisense strand sequencing respectively. PCR conditions for each gene is shown, size of PCR product and position of methylation assay relative to transcription start site (TSS).
