# Supplementary material for: Sex differences in saliva-based DNA methylation changes and environmental stressor in young African American adults
Source: PLoS One. 2022 Sep 6;17(9):e0273717. doi: 10.1371/journal.pone.0273717 (PMC9447871; doi:10.1371/journal.pone.0273717)
Supplement: S2 Table — (DOCX) [file pone.0273717.s003.docx]

**Table S2**

Descriptive characteristic of participants (N=98)

| Variables |  | Mean (SD) or percent |
| --- | --- | --- |
| *Age 18-25* |  | 20.4 (1.9) |
| **Importance of religion/spirituality**  Not important  Somehow important  Very important |  | 19.4%  33.7%  46.9% |
| **Gende**r  Female  Male |  | 51.0%  49.9% |
|  |  |  |
| **Source of household income**  People who worked  Welfare or public assistance  Worked and welfare  Other |  | 65.3%  19.4%  11.2%  4.1% |
| **I Currently live in**  A place I rent  A place I own  Public housing  A place where I don’t have to pay anything  Ever drank alcohol (yes)  Ever smoked cigarette (yes)  Ever used marijuana (yes) |  | 33.8%  11.3%  26.3%  28.8%  77.6%  48.0%  59.2% |
|  |  |  |
|  |  |  |

Descriptive characteristics and socioeconomic status variables of participants are summarized in Table S2. The mean age of the participants was 20.4 +1.9. About 65% source of household income was from work. 11.3% of the participants owned their place of residence. 77.6% of participants had at least one drink during their lifetime, 48% had ever smoked a cigarette and 59.2% had ever used marijuana.
